# HDL subfractions and very early CAD: novel findings from untreated patients in a Chinese cohort

**DOI:** 10.1038/srep30741

**Published:** 2016-08-04

**Authors:** Yan Zhang, Cheng-Gang Zhu, Rui-Xia Xu, Sha Li, Xiao-Lin Li, Yuan-Lin Guo, Na-Qiong Wu, Ying Gao, Ping Qing, Chuan-Jue Cui, Jing Sun, Jian-Jun Li

**Affiliations:** 1 Division of Dyslipidemia, State Key Laboratory of Cardiovascular Disease, Fu Wai Hospital, National Center for Cardiovascular Diseases, Chinese Academy of Medical Sciences, Peking Union Medical College, BeiLiShi Road 167, Beijing 100037, China

## Abstract

Coronary artery disease (CAD) in very young individuals is a rare disease associated with poor prognosis. However, the role of specific lipoprotein subfractions in very young CAD patients (≤45 years) is not established yet. A total of 734 consecutive CAD subjects were enrolled and were classified as very early (n = 81, ≤45), early (n = 304, male: 45–55; female: 45–65), and late (n = 349, male: >55; female: >65) groups. Meanwhile, a group of non-CAD subjects were also enrolled as controls (n = 56, ≤45). The lipoprotein separation was performed using Lipoprint System. As a result, the very early CAD patients have lower large high-density lipoprotein (HDL) subfraction and higher small low-density lipoprotein (LDL) subfraction (p < 0.05). Although body mass index was inversely related to large HDL subfraction, overweight did not influence its association with very early CAD. In the logistic regression analysis, large HDL was inversely [OR 95% CI: 0.872 (0.825–0.922)] while small LDL was positively [1.038 (1.008–1.069)] related to very early CAD. However, after adjusting potential confounders, the association was only significant for large HDL [0.899 (0.848–0.954)]. This study firstly demonstrated that large HDL subfraction was negatively related to very early CAD suggestive of its important role in very early CAD incidence.

The prevalence of coronary artery disease (CAD) has increased sharply and manifested a younger trend, which has becoming an important public health issue^1^. Although it has been estimated that less than 10% of all individuals presenting with documented CAD are in very young ages, it can have devastating consequences for these patients, their families, and society due to the high morbidity and long-term mortality^2^.

Till now, the extent of clinical risk factors for CAD occurrence in the young population has been difficult to determine. In terms of traditional risk factors, there is no unique one present in large groups of young adults with CAD^3^. Previous epidemiological studies indicated that the relatively more important risk factors in young patients are their elevated body mass index (BMI), smoking habits, hypertension, and specifically, dyslipidemia^4^. Currently, the treatment of dyslipidemia has been established as one of the principal targets in clinical practice due to its key role in the development of CAD^5^,^6^. However, despite the major advances in the treatment of dyslipidemia, such as the low-density lipoprotein (LDL) cholesterol (LDL-C) lowering^7^,^8^ and high-density lipoprotein (HDL) cholesterol (HDL-C) raising^9^,^10^ strategies, residual cardiovascular risk remains high in a significant number of patients^11^. Promisingly, recent studies demonstrated that the cholesterol content of LDL or HDL particles displays a large inter-individual variation^12^,^13^.

Although the dysfunction of lipid metabolism is a major contributor for CAD development and progression, lipoprotein subfractions have been suggested to be more precisely reflecting the atherogenity of lipids. Recently, our group demonstrated that patients with CAD have relatively lower large HDL subfraction and higher small HDL and LDL subfraction, providing new perspectives with regard to the role of different lipoprotein subfractions in the CAD prevalence^14^. In light of the specialization of patients with CAD in young ages, we hypothesized that the distribution and impact of lipoprotein subfractions in younger CAD patients may be varied with those in older ones. However, such data has been unavailable till now.

Therefore, the aim of the present study was to compare LDL and HDL subfractions separated by Lipoprint System among controls without CAD (≤45), very early CAD (≤45), early (male: 45–55; female: 45–65), and late CAD (male: >55; female: >65) patients. Furthermore, we also aimed to assess the influence of different lipoprotein subfractions on very early CAD (≤45 years of age) susceptibility.

## Methods

### Study design and population

The study complied with the Declaration of Helsinki and was approved by the hospital’s ethical review board (FuWai Hospital & National Center for Cardiovascular Diseases, Beijing, China). Each participant provided written, informed consent before enrollment.

From October 2012 to June 2015, we consecutively recruited 734 patients with angiography proven CAD and a total of 56 non-CAD controls (≤45 years of age) in our institution. All the enrolled CAD patients were classified into three groups: very early CAD (≤45 years of age, n = 81), early CAD (male: 45–55 years of age; female: 45–65 years of age, n = 304), and late CAD (male: >55 years of age; female: >65 years of age, n = 349) groups. Considering the potential influence of lipid lowering drugs on plasma levels of lipid profiles as well as lipoprotein subfractions, we only included patients who were not on the treatment of statins and/or other lipid-lowering drugs at least 3 months before entering the study. Exclusion criteria were subjects over 90 years, pregnancy or lactation, psychiatric disorder, the existence of any infectious or systematic inflammatory disease within 1 month, acute coronary syndrome, serious heart failure or arrhythmia, significant hematologic disorders, thyroid dysfunction, severe liver dysfunction (aspartate aminotransperase or alanine aminotrabsferase three times more than the upper normal limits) and/or renal insufficiency (blood creatinine > 1.5 mg/dL) and malignant tumors.

As depicted in our previous studies^15^, the traditional risk factors were defined as follows. Hypertension was defined as repeated blood pressure measurements ≥140/90 mmHg (at least two times in different environments) or self-reported hypertension and currently taking anti-hypertensive drugs. Diabetes mellitus (DM) was defined as a fasting serum glucose level ≥126 mg/dL in multiple determinations, and/or the current use of medication for diabetes. Dyslipidemia was defined by medical history or fasting total cholesterol (TC) ≥200 mg/dL or triglyceride (TG) ≥150 mg/dL. BMI was calculated as weight (kg) divided by height (m) squared. Overweight was defined as BMI ≥ 25 kg/m^2^.

### Biochemical and clinical analyses

Fasting blood samples were collected in pre-cooled EDTA tubes at baseline from each patient. After centrifugation at 3000 rpm for 15 min at 4 °C, all plasma aliquots were stored in our laboratory at −80 °C and were not thawed until use. The plasma levels of LDL-C and HDL-C were analyzed directly by selective solubilization method (Low density lipid cholesterol test kit or Determiner L HDL, Kyowa Medex, Tokyo). TC and TG were measured by enzymatic methods. All of the lipid profiles were determined using automatic biochemistry analyzer (Hitachi 7150, Tokyo, Japan).

### LDL and HDL subfraction analysis

The cholesterol contents of LDL and HDL subfractions were determined electrophoretically by the Lipoprint System (Lipoprint LDL System and Lipoprint HDL System, respectively; Quantimetrix Corporation, Redondo Beach, CA, USA) according to the manufacturer’s instructions as described elsewhere^16^,^17^. This method was based on electrophoresis of a liquid loading gel with lipophilic dye in the precast linear polyacrylamide gel (stacking gel and separating gel). For LDL particle, a typical Lipoprint profile of decreasing size and increasing density with 1 very low density lipoprotein (VLDL) band, 3 Midbands, up to 7 LDL bands, and 1 HDL band were obtained. The various stained bands (lipoprotein subfractions) presented in the sample were identified by their electrophoretic mobility (Rf) using VLDL as the starting reference point (Rf = 0) and HDL as the leading reference point (Rf = 1). Seven LDL subfractions were obtained. Subfraction 1 represented large LDL particles, subfraction 2 indicated medium LDL particles, and subfractions 3–7 were defined as small dense LDL particles. Similarly, for HDL particle, the Lipoprint HDL system using VLDL/LDL as the starting reference point (Rf = 0) and albumin as the leading reference point (Rf = 1). Between the two points, 10 HDL subfractions were obtained. Subfractions 1–3 represented large HDL particles, subfractions 4–7 indicated medium HDL particles, and subfractions 8–10 meant small HDL particles. The cholesterol concentration (mg/dL) of each lipoprotein subfraction and the mean LDL particle size (Å) were determined by this assay.

### Statistical analysis

The data were expressed as the mean ± SD for the continuous variables and the number (percentage) for the categorical variables. The student t test, one-way analysis of variance, or non-parametric test was used for the comparison between/among groups of continuous parameters as appropriate. The categorical variables were compared using the chi-square test. Multivariate logistic regression analysis was used for determining the association of LDL or HDL subfractions with the incident of very early CAD susceptibility. A p value of less than 0.05 was considered statistically significant. Statistical studies were carried out with the SPSS program (version 19.0, SPSS, Chicago, Illinois, USA).

## Results

### Summary of Study Subjects

The baseline demographics and clinical characteristics of the study population at baseline were shown in Table 1. Overall, the enrolled subjects were classified into four groups according to the presence of CAD and the age of CAD onset. Significantly, compared with the non-CAD controls (with a mean age of 41.1 ± 2.9 years old), the very early CAD patients have higher BMI levels, higher percentage of hypertension and dyslipidemia. Meanwhile, in comparison with the relatively older CAD patients, the very early cases were more likely to be more male gender, to have a higher BMI level, diastolic blood pressure, current smokers, and family history of CAD. However, the levels of inflammatory markers such as white blood cell count, fibrinogen, and high-sensitivity C reactive protein (all p > 0.05) were similar among groups except for the lower concentrations of D-dimer (p < 0.001) and erythrocyte sedimentation rate (p = 0.004).

The angiographic characteristics of CAD participants according to age were presented in Table 2. The left anterior descending artery was less frequently involved (86.1% vs. 87.2% vs. 92.9%, p = 0.030) while the other related arteries were similar (p > 0.05) in the very early patients compared with early and late cases. In addition, compared to the early and late CAD patients, the very early cases have higher percentage of single artery disease (39.0% vs. 31.4% vs. 24.6%, p = 0.023) and less total number of diseased vessels (2.04 ± 0.98 vs. 2.15 ± 0.96 vs. 2.34 ± 0.95, p = 0.009).

### HDL and LDL subfractions in very early CAD patients

**Figure 1:**
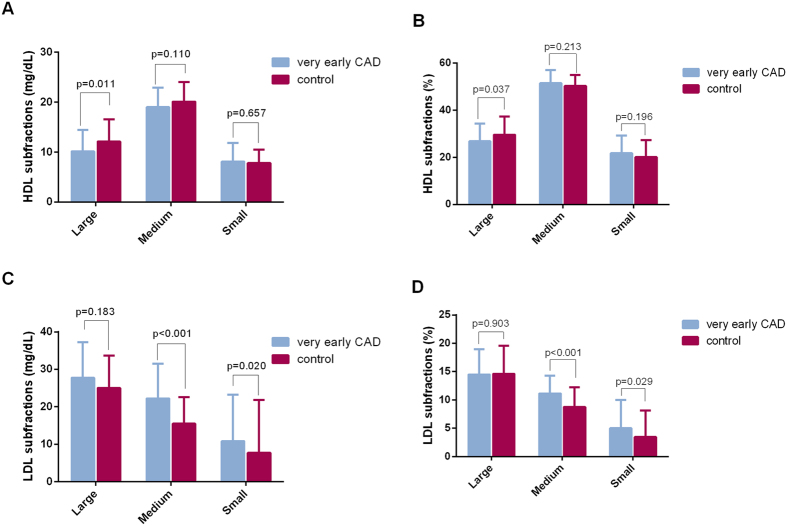
The comparison of HDL (**A**,**B**) and LDL (**C**,**D**) subfractions between very early CAD and controls. Student t test or non-parametric test was applied as appropriate.

To exclude the potential impact of age on the distribution of lipoprotein subfractions, we analyzed these parameters in very young patients with and without CAD (≤45 years of age). We finally observed that the very early CAD patients have relatively lower large HDL and higher medium and small LDL subfractions (p < 0.05, all) (Fig. 1). Moreover, by contrast to early and old CAD patients, as shown in Table 3, the very early CAD cases have higher mean concentrations of TG, TC, and LDL-C but lower levels of HDL-C (all p < 0.01). Regarding to the lipoprotein subfraction analysis, we found that the concentrations of large and medium HDL subfraction (both cholesterol levels and percentages) were significantly lower in the very early CAD group (cholesterol levels of large HDL: 10.16 ± 4.31 vs. 12.47 ± 6.23 vs. 14.34 ± 7.30 mg/dL, p < 0.001; large HDL percentage: 26.77 ± 7.52 vs. 29.10 ± 8.03 vs. 32.04 ± 7.99%, p < 0.001; cholesterol levels of medium HDL: 19.01 ± 3.93 vs. 20.75 ± 6.57 vs. 21.01 ± 6.44 mg/dL, p = 0.035; medium HDL percentage: 51.42 ± 5.58 vs. 50.04 ± 5.17 vs. 48.95 ± 4.80%, p < 0.001). On the contrary, the concentrations of small HDL subfraction (percentage) were significantly higher in the very early CAD group (21.75 ± 7.47 vs. 20.63 ± 7.54 vs. 18.95 ± 6.22%, p < 0.001). Meanwhile, the very early CAD group has markedly higher medium LDL subfraction (cholesterol levels: 22.24 ± 9.23 vs. 20.77 ± 8.95 vs. 19.20 ± 8.98 mg/dL, p = 0.039) and small LDL subfraction (cholesterol levels: 10.80 ± 12.41 vs. 10.27 ± 10.00 vs. 7.45 ± 8.33 mg/dL, p = 0.003; percentages: 4.99 ± 4.97 vs. 5.02 ± 4.51 vs. 3.79 ± 3.93%, p = 0.008) as well as smaller mean LDL particle size (265.54 ± 6.13 vs. 265.42 ± 5.93 vs. 267.03 ± 5.54 Å, p = 0.011).

**Figure 2:**
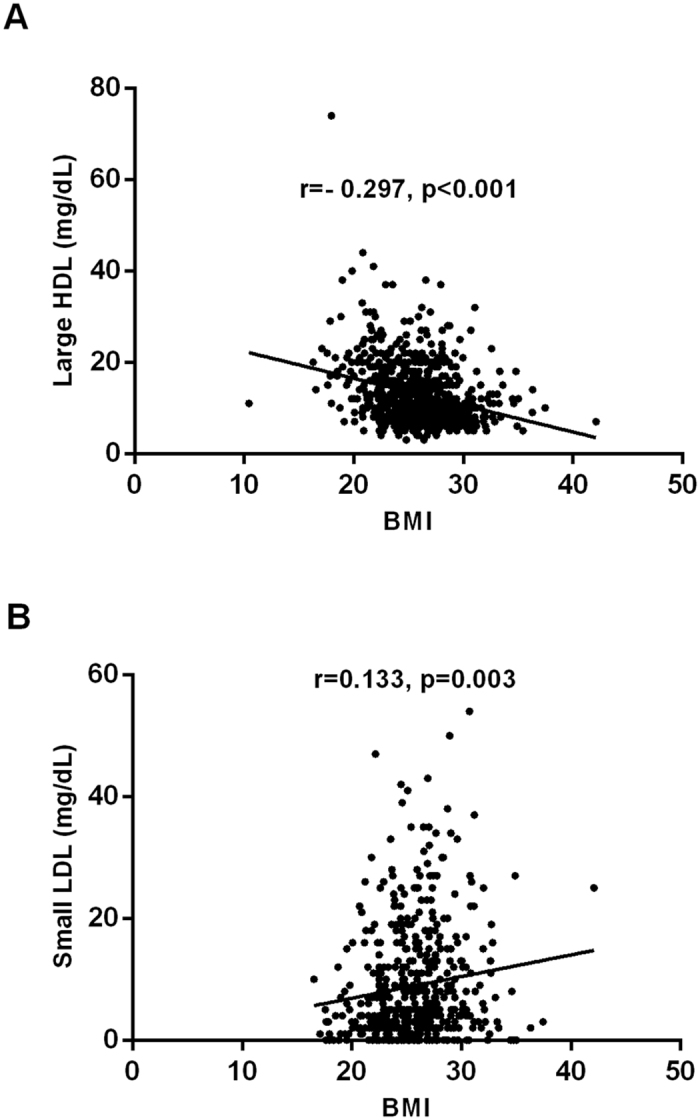
The relationship between BMI and large HDL-C (**A**) or small LDL-C (**B**). Pearson correlation analysis was applied.

**Figure 3:**
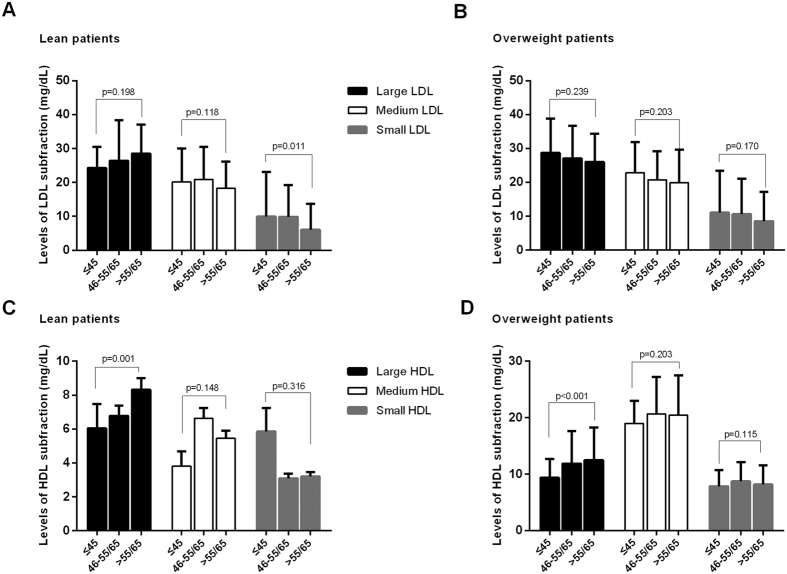
The association of LDL (**A**,**B**) or HDL (**C**,**D**) subfraction stratified by overweight. ANOVA was used in the current analysis.

In the current analysis, the very early CAD patients have significantly high BMI levels. Specifically, we found that BMI was negatively associated with cholesterol levels of large HDL (r = −0.297, p < 0.001, Fig. 2A) while positively related to cholesterol levels of small LDL (r = 0.133, p = 0.003, Fig. 2B). Next, we further investigated the differences in lipoprotein subfractions by comparing the lean (BMI < 25 kg/m^2^) and the overweight CAD patients (BMI ≥ 25 kg/m^2^). As a result, the cholesterol levels of small LDL was highest in the very early CAD group only in the lean but not in the overweight cases (Fig. 3A,B) while the cholesterol levels of large HDL was lowest both in the lean and overweight cases (Fig. 3C,D).

### Relation of lipoprotein subfractions to very early CAD incidence

After observed the association of lipoprotein subfractions with very early CAD cases, logistic regression analysis was performed in the current study. In unadjusted analysis (Table 4), among different HDL subfractions, large and medium HDL measures were inversely [OR 95%CI: large HDL: 0.872 (0.825–0.922); medium HDL: 0.935 (0.889–0.983)] associated with the incident of very early CAD. Therefore, in the following multivariate logistic regression analysis, we further adjusted for BMI as well as other potential risk factors covering sex, hypertension, dyslipidemia, DM, current smoking, and family history of CAD. We finally found that only the cholesterol levels of large HDL [OR 95%CI: 0.899 (0.848–0.954)] remained negatively related to very early CAD susceptibility (Table 4).

## Discussion

The current study is the first to document the relationship between lipoprotein subfractions and very early CAD occurrence involving 734 consecutive CAD patients and 56 non-CAD controls who were not treated with lipid-lowering drugs. Specifically, we found that CAD patients in younger ages have significantly lower large HDL subfractions, higher small HDL and LDL subfractions, and relatively smaller mean LDL particle size. In the logistic regression analysis, large HDL subfraction was associated with lower risk while small LDL subfraction was related to higher risk of very early CAD. In addition, we found that overweight was not only related to large HDL and small LDL subfraction but also the age of CAD incidence. However, only large HDL subfraction remained negatively associated with CAD in younger ages after adjusting for BMI and other potential confounders. Our data may provide novel information with regard to the potential role of different lipoprotein subfractions in the incident of very early CAD.

Although the CAD occurrence in very young ages has a relatively low prevalence rate, it can have devastating consequences. During the past decades, multiple studies have tried to address the issue why it happens in these very young individuals^18^ and finally emphasized BMI, smoking habits, hypertension, family history of CAD, and dyslipidemia as more relevant risk factors^4^. However, it has been difficult to define risk factors unique to this population because all of these are traditional risk factors for common CAD patients. Undoubtedly, the elevated lipid and lipoprotein levels remain one of the most pivotal risk factors for the development of CAD in young ages. Previous study has revealed that the role of higher TC and LDL-C and lower HDL-C levels appeared to be important factors in the process of very early CAD process^19^,^20^. However, the concept of lipoprotein particle or subfraction has recently challenged the relevance of the cholesterol content of lipoproteins^13^,^21^. The small dense LDL-C has been demonstrated to be associated with the incident CAD in the Atherosclerosis Risk in Communities study involving 11,419 participants^22^. A variety of mechanisms have been proposed to explain the enhanced atherogenicity of small dense LDL, such as the higher penetration into the arterial wall, prolonged plasma half-life, and lower affinity for the LDL-receptor^23^. Recently, Martin *et al*. reported that low HDL3-C (small HDL-C) subclasses, but not HDL2-C (large HDL-C) was associated with increased long-term hard clinical events in two cohorts of secondary prevention^24^. In our recent study involving 591 un-treated patients, large HDL has been proven to be associated with lower rate of future cardiovascular events^25^. Till now, the relationship between HDL subfraction and cardiovascular risk remains in debate and the potential mechanisms have not been elucidated yet. In light of the preceding discussion, we tentatively investigate the distribution and potential impact of LDL and HDL subfractions with very early CAD presence.

Actually, LDL and HDL particles are comprised of a variety of different subfractions that can be separated by several methods. Among the various lipoprotein separation methods, Lipoprint system, nuclear magnetic resonance (NMR) spectroscopy, and Vertical Auto Profile method (VAP) are most commonly applied in clinical research. In this study, we applied the Lipoprint system and 10 HDL and 7 LDL subclasses (large HDL: 1–3, medium HDL: 4–7, and small HDL: 8–10; large LDL: 1; medium LDL: 2; small LDL: 3–7) have been separated. This method was based on decreasing size and increasing density by electrophoresis of a liquid loading gel with lipophilic dye in the precast linear polyacrylamide gel (stacking gel and separating gel)^26^. In addition, NMR was the currently common used method in clinical and laboratory research. In this way, HDL and LDL subclasses were quantified using the amplitudes of their spectroscopically distinct lipid methyl group NMR signals^27^. The VAP separates lipoproteins on the base of density using single vertical-spin density gradient ultracentrifugation^24^. The existing of diverse lipoprotein separation methodologies may mainly contribute to discrepancies. As early in 1991, Salonen *et al*. reported that large HDL-C levels were inversely associated with the risk of acute myocardial infarction and may thus be protective factors^28^. Besides that, our recent studies have revealed that large HDL subfraction was negatively associated with several cardiovascular risk factors, such as serum uric acid^29^ and hypertension^30^. In the current study, we found that only large HDL subfraction was negatively associated with very early CAD susceptibility, which may provide additive information regarding the different role of specific subfraction on the very early CAD incidence.

There were several limitations of the present study. First, the cross-sectional design was a limitation. Therefore, the results should be evaluated with some degree of caution. Second, this was a single center study with relatively small sample size (the very early CAD group, n = 81), the data should be confirmed by large scale studies. Finally, the lipoprotein subclassification was performed by Lipoprint system, NMR and other methodologies may be necessary in the future studies.

In summary, the CAD patients in younger ages have relatively lower large HDL subfractions, smaller mean LDL particle size, and higher small HDL and LDL subfractions. Only large HDL subfraction was inversely and independently associated with incident of very early CAD after adjusting for potential confounders. Our data for the first time revealed the distribution of lipoprotein subfractions in very early CAD status, suggesting the potential role of large HDL subfraction in the very early CAD susceptibility.

## Additional Information

**How to cite this article**: Zhang, Y. *et al*. HDL subfractions and very early CAD: novel findings from untreated patients in a Chinese cohort. *Sci. Rep.* **6**, 30741; doi: 10.1038/srep30741 (2016).

## Figures and Tables

**Table 1:** Clinical and biochemical characteristics.

	Control	Very early	Early	Late	p value^a^	p value^b^
	n = 56	n = 81	n = 304	n = 349		
**Risk factors**						
Age (year)	41.1 ± 2.9	41.3 ± 3.5	54.0 ± 5.1	65.3 ± 6.7	0.737	**<0.001**
Male, n (%)	46 (82.1)	73 (90.1)	167 (54.9)	284 (81.4)	0.204	**<0.001**
BMI (kg/m^2^)	25.7 ± 4.1	27.1 ± 3.5	25.7 ± 3.2	25.5 ± 3.5	**0.026**	**<0.001**
SBP (mm Hg)	120.3 ± 15.3	121.8 ± 12.5	129.0 ± 16.8	131.0 ± 17.7	0.547	**<0.001**
DBP (mm Hg)	78.9 ± 10.7	80.5 ± 12.0	80.5 ± 11.4	77.8 ± 11.1	0.421	**0.005**
Smoking, n (%)	27 (48.2)	39 (48.1)	117 (38.5)	125 (35.8)	0.122	**<0.001**
Hypertension, n (%)	16 (28.6)	46 (56.8)	193 (63.5)	233 (66.8)	**0.002**	0.223
DM, n (%)	7 (12.5)	16 (19.8)	75 (24.7)	105 (30.1)	0.354	0.096
Dyslipidemia, n (%)	28 (50.0)	57 (70.4)	184 (60.5)	204 (58.5)	**0.020**	0.141
Family history, n (%)	9 (16.1)	20 (24.7)	74 (24.3)	49 (14.0)	0.289	**0.002**
**Laboratory and clinical test**						
D-dimer (μg/mL)	0.26 ± 0.16	0.27 ± 0.15	0.35 ± 0.32	0.43 ± 0.40	0.816	**<0.001**
WBC count (10^9^/L)	6.45 ± 1.89	6.58 ± 1.76	6.29 ± 1.67	6.24 ± 1.73	0.682	0.266
ESR (mm/h)	4.5 (2–10)	5 (2–8)	7 (3–13)	7 (3–13)	0.825	**0.004**
hs-CRP (mg/L)	1.07 (0.64–2.19)	1.37 (0.68–2.69)	1.49 (0.75–2.99)	1.59 (0.68–3.31)	0.313	0.773
Fibrinogen (g/L)	2.86 ± 0.65	3.04 ± 0.77	3.09 ± 0.78	3.18 ± 0.84	0.150	0.223
LVEF (%)	67.1 ± 5.4	65.8 ± 7.3	64.9 ± 7.4	64.8 ± 7.6	0.251	0.558
**Prior drug treatment**						
Aspirin, % (n)	10 (17.9)	32 (39.5)	123 (40.5)	151 (43.3)	**0.008**	0.703
Beta-blocker, % (n)	8 (14.3)	19 (23.5)	60 (19.7)	79 (22.6)	0.199	0.604
ACEI, % (n)	1 (1.8)	3 (3.7)	17 (5.6)	33 (9.5)	0.645	0.071
ARB, % (n)	2 (3.6)	9 (11.1)	27 (8.9)	48 (13.8)	0.199	0.148
CCB, % (n)	5 (8.9)	12 (14.8)	65 (21.4)	88 (25.2)	0.430	0.109

Data are expressed as mean ± SD or n (%). The bold values indicated statistical significance. BMI, body mass index; SBP, systolic blood pressure; DBP, diastolic blood pressure; DM, diabetes mellitus; WBC, white blood cell; ESR, erythrocyte sedimentation rate; hs-CRP, high sensitivity C-reactive protein; LVEF, left ventricular ejection fraction; ACEI, angiotensin converting enzyme inhibitor; ARB, angiotensin receptor blocker; CCB, calcium channel blocker. p value^a^ for very early CAD vs. control group. p value^b^ for very early CAD vs. early vs. late group.

**Table 2:** Angiographic characteristics of CAD participants according to ages.

	Very early	Early	Late	p value
	n = 81	n = 304	n = 349	
**Involved stenotic coronary arteries**				
LM-diseased	9 (11.4)	47 (15.4)	52 (15.0)	0.659
LAD-diseased	70 (86.1)	265 (87.2)	324 (92.9)	**0.030**
LCX-diseased	43 (53.2)	181 (59.4)	230 (65.9)	0.060
RCA-diseased	44 (54.4)	173 (57.0)	217 (62.1)	0.292
**Number of stenotic coronaryarteries**				**0.023**
1-vessel diseased	32 (39.0)	95 (31.4)	86 (24.6)	
2-vessels diseased	20 (24.7)	92 (30.1)	93 (26.6)	
Multi-vessels diseased	29 (36.4)	117 (38.5)	170 (48.8)	
**Total number of stenotic vessels**	2.04 ± 0.98	2.15 ± 0.96	2.34 ± 0.95	**0.009**
**Gensini score**	22 (10.5–44)	24 (10–52)	24 (12–58)	0.161

Data are expressed as mean ± SD or n (%). The bold values indicated statistical significance. LM, left main coronary artery; LAD, left anterior descending artery; LCX, left circumflex artery; RCA, right coronary artery.

**Table 3:** Lipoprotein subfractions in CAD participants according to ages.

	Very early	Early	Late	p value
	n = 81	n = 304	n = 349	
**Lipid parameters**				
TG (mg/dL)	191.2 ± 105.0	176.0 ± 112.4	152.5 ± 85.1	**0.001**
TC (mg/dL)	193.3 ± 42.5	192.7 ± 41.8	182.5 ± 36.1	**0.002**
HDL-C (mg/dL)	36.80 ± 7.87	41.82 ± 12.45	43.01 ± 13.58	**<0.001**
LDL-C (mg/dL)	127.42 ± 42.02	128.56 ± 38.05	118.94 ± 34.25	**0.001**
**HDL subfraction**				
Large HDL (mg/dL)	10.16 ± 4.31	12.47 ± 6.23	14.34 ± 7.30	**<0.001**
Medium HDL (mg/dL)	19.01 ± 3.93	20.75 ± 6.57	21.01 ± 6.44	**0.035**
Small HDL (mg/dL)	8.10 ± 3.74	8.35 ± 3.33	8.01 ± 3.29	0.435
Large HDL (%)	26.77 ± 7.52	29.10 ± 8.03	32.04 ± 7.99	**<0.001**
Medium HDL (%)	51.42 ± 5.58	50.04 ± 5.17	48.95 ± 4.80	**<0.001**
Small HDL (%)	21.75 ± 7.47	20.63 ± 7.54	18.95 ± 6.22	**<0.001**
**LDL subfraction**				
Large LDL (mg/dL)	27.70 ± 9.52	26.87 ± 10.65	27.11 ± 8.46	0.846
Medium LDL (mg/dL)	22.24 ± 9.23	20.77 ± 8.95	19.20 ± 8.98	**0.039**
Small LDL (mg/dL)	10.80 ± 12.41	10.27 ± 10.00	7.45 ± 8.33	**0.003**
Large LDL (%)	14.49 ± 4.46	13.86 ± 4.23	14.75 ± 3.90	0.076
Medium LDL (%)	11.14 ± 3.12	10.50 ± 3.24	10.13 ± 3.70	0.130
Small LDL (%)	4.99 ± 4.97	5.02 ± 4.51	3.79 ± 3.93	**0.008**
Mean LDL particle size (Å)	265.54 ± 6.13	265.42 ± 5.93	267.03 ± 5.54	**0.011**

Data are expressed as mean ± SD. The bold values indicated statistical significance. TG, triglyceride; TC, total cholesterol; HDL-C, high-density lipoprotein-cholesterol; LDL-C, low-density lipoprotein-cholesterol; HDL, high-density lipoprotein; LDL, low-density lipoprotein.

**Table 4:** Relation of lipoprotein subfraction with prevalent of very early CAD.

	Late	Early	p value	Very early	p value
**Unadjusted analysis**					
**HDL subfraction** (mg/dL)					
Large HDL	1	0.958 (0.934–0.982)	**0.001**	0.872 (0.825–0.922)	**<0.001**
Medium HDL	1	0.994 (0.970–1.018)	0.604	0.935 (0.889–0.983)	**0.008**
Small HDL	1	1.030 (0.984–1.079)	0.203	1.008 (0.937–1.086)	0.827
**LDL subfraction** (mg/dL)					
Large LDL	1	0.997 (0.977–1.017)	0.743	1.006 (0.976–1.037)	0.682
Medium LDL	1	1.021 (0.999–1.043)	0.057	1.037 (1.005–1.071)	**0.024**
Small LDL	1	1.033 (1.012–1.055)	**0.002**	1.038 (1.008–1.069)	**0.013**
**Mean LDL particle size (Å)**	1	0.951 (0.919–0.983)	**0.003**	0.955 (0.908–1.004)	0.072
**Adjusted analysis**					
**HDL subfraction** (mg/dL)					
Large HDL (mg/dL)	1	0.933 (0.905–0.961)	**<0.001**	0.899 (0.848–0.954)	**<0.001**
Medium HDL (mg/dL)	1	0.985 (0.959–1.012)	0.274	0.956 (0.908–1.007)	0.091
Small HDL (mg/dL)	1	1.015 (0.966–1.067)	0.552	0.992 (0.918–1.072)	0.839
**LDL subfraction** (mg/dL)					
Large LDL	1	0.989 (0.968–1.011)	0.335	1.001 (0.968–1.036)	0.940
Medium LDL	1	1.012 (0.988–1.037)	0.327	1.014 (0.979–1.051)	0.425
Small LDL	1	1.028 (1.005–1.052)	**0.016**	1.021 (0.990–1.054)	0.184
**Mean LDL particle size (Å)**	1	0.954 (0.920–0.990)	**0.012**	0.973 (0.921–1.028)	0.323

Logistic regression analysis was applied. The bold values indicated statistical significance. HDL, high-density lipoprotein; LDL, low-density lipoprotein. The adjusted covariates included sex, BMI, hypertension, dyslipidemia, DM, current smoking, and family history of CAD.
